# Using hidden Markov models to investigate G-quadruplex motifs in genomic sequences

**DOI:** 10.1186/1471-2164-15-S9-S15

**Published:** 2014-12-08

**Authors:** Masato Yano, Yuki Kato

**Affiliations:** 1 Graduate School of Information Science, Nara Institute of Science and Technology (NAIST), 8916-5 Takayama, Ikoma, Nara 630-0192, Japan; 2 Center for iPS Cell Research and Application (CiRA), Kyoto University, 53 Kawahara-cho, Shogoin, Sakyo-ku, Kyoto 606-8507, Japan

## Abstract

**Background:**

G-quadruplexes are four-stranded structures formed in guanine-rich nucleotide sequences. Several functional roles of DNA G-quadruplexes have so far been investigated, where their putative functional roles during DNA replication and transcription have been suggested. A necessary condition for G-quadruplex formation is the presence of four regions of tandem guanines called G-runs and three nucleotide subsequences called loops that connect G-runs. A simple computational way to detect potential G-quadruplex regions in a given genomic sequence is pattern matching with regular expression. Although many putative G-quadruplex motifs can be found in most genomes by the regular expression-based approach, the majority of these sequences are unlikely to form G-quadruplexes because they are unstable as compared with canonical double helix structures.

**Results:**

Here we present elaborate computational models for representing DNA G-quadruplex motifs using hidden Markov models (HMMs). Use of HMMs enables us to evaluate G-quadruplex motifs quantitatively by a probabilistic measure. In addition, the parameters of HMMs can be trained by using experimentally verified data. Computational experiments in discriminating between positive and negative G-quadruplex sequences as well as reducing putative G-quadruplexes in the human genome were carried out, indicating that HMM-based models can discern bona fide G-quadruplex structures well and one of them has the possibility of reducing false positive G-quadruplexes predicted by existing regular expression-based methods. Furthermore, our results show that one of our models can be specialized to detect G-quadruplex sequences whose functional roles are expected to be involved in DNA transcription.

**Conclusions:**

The HMM-based method along with the conventional pattern matching approach can contribute to reducing costly and laborious wet-lab experiments to perform functional analysis on a given set of potential G-quadruplexes of interest. The C++ and Perl programs are available at http://tcs.cira.kyoto-u.ac.jp/~ykato/program/g4hmm/.

## Background

Deoxyribonucleic acids (DNAs) are macromolecules that hold genetic information in almost all of the organisms. The bulk of existing DNA molecules is assumed to form a right-handed double helical structure called B-DNA [1], where each constituent bases A and C selectively bind to bases T and G, respectively, between two strands arranged in the antiparallel way. In contrast, several in vitro experiments reveal the existence of non-B-DNA structures caused by particular sequence motifs and DNA-protein interactions. Well investigated examples include *G-quadruplex *(G4), Z-DNA, cruciform and triplex. Recent advances in providing in vitro evidence of these specific structures develop the hypothesis that these structures are considered to have some functional roles in living cells [2].

**Figure 1:**
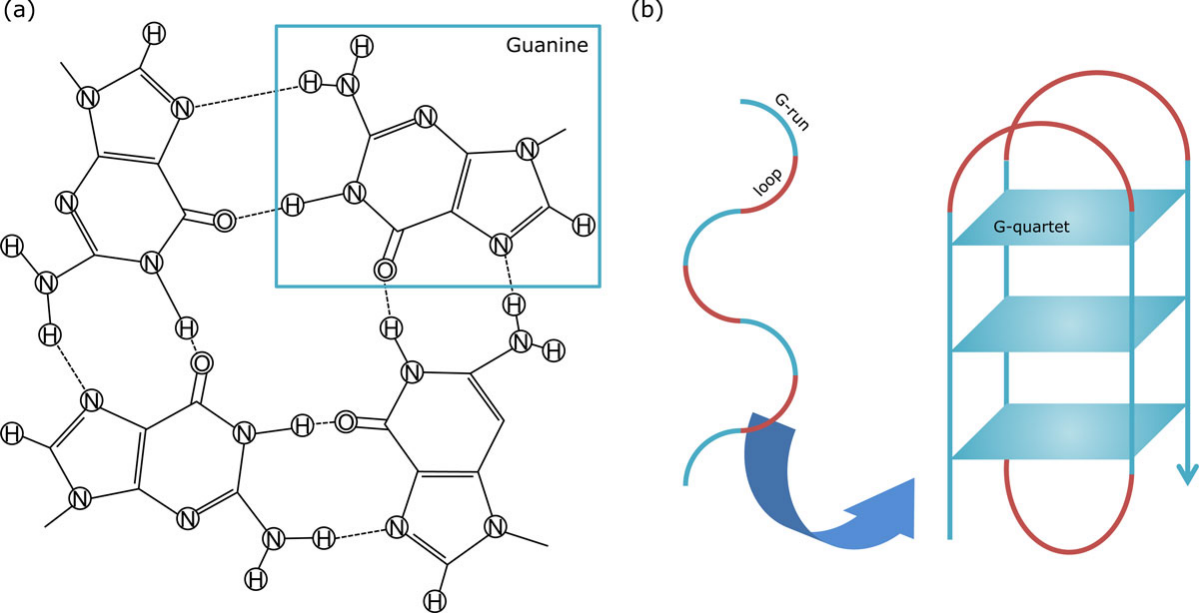
**An example of G4 structure**. (a) Four guanines form hydrogen bonds to their adjacent guanines, resulting in a G-quartet. (b) Several G-quartets are stacked onto each other to form a G-quadruplex (G4) structure. A G4 structure has its sequence as alternate G-runs and loops.

A G4 structure is one of the topological conformations that DNAs can adopt, where G-quartets, hydrogen-bonded square planar substructures between four guanines (Gs), are stacked onto each other (see Figure 1). At the sequence level, a G4 sequence can be represented by four regions of consecutive Gs that form G-quartets, called *G-runs*, and three regions of nucleotide subsequences that connect G-runs, called *loops*, which can have varying length including lack of loop [3]. Note that consecutive Gs may stretch over G-run and loop regions, making the problem of predicting G-run regions somewhat complex. Loops are known to play an important role in stability of G4 structures [4]. In addition, it is pointed out that the loop length affects therapeutic selectivity to target a G4 instead of the topology of the G4 structure [5]. G4 structures are also stabilized by monovalent cations, especially K^+^, located in the central cavities in the stack.

Eukyariotic telomeric sequences include G-rich regions and they can form G4 structures in vitro. However, the question of how many such G-rich regions can actually form G4 structures in vivo has not been resolved. The potential to form G4 structures in telomeric sequences in vivo can be shown by in vitro DNA binding experiments with those sequences [2]. For example, telomere end-binding proteins in ciliates can control the formation of G4 DNA structures at telomeres [6]. Interestingly, however, a recent study suggests that endogenous G4 structures in human cells are present largely outside the telomeres [7]. Another work reports that protruding nucleotides in human telomeric sequences destabilize the G4 structure and overhanging sequences influence the folding of the quadruplex [8]. Other examples of G-rich regions in genomes are transcriptional start sites, mitotic and meiotic double strand break sites. Although G4 structures have stability with higher temperature than that of canonical double helix structures, many functional regions in genomic sequences have not a few G-rich motifs [2], motivating us to investigate further the functional roles of G4 structures.

Since little is known about the functions of G4 structures and genome-scale wet-lab experiments with nuclear magnetic resonance (NMR) spectroscopy for structural analysis [9] are not feasible, several computational efforts have been made on identifying the locations of potential G4 sequences in genomic DNAs and inferring their functions by comparative sequence analysis using related genes with known functions [10,11]. In principle, G4 motifs can be represented by a regular expression *G*^+^*N *^∗^*G*^+^*N*^∗^*G*^+^*N*^∗^*G*^+^, where '*N*' shows an arbitrary base including G, '+' denotes at least one repeat of the preceding symbol and '∗' means at least zero repeats. Due to this simple pattern of G4 motifs, several in silico methods have been proposed to detect G4 sequences in genomes using pattern matching with regular expression [12-16]. Moreover, regular expression-based methods that incorporate a simple scoring scheme are proposed [17-19]. Another computational study focuses on thermodynamic stability of G4 structures using Gaussian process regression [20]. Although the pattern matching approaches can detect many G4 motifs in genomic sequences quite fast, it is pointed out that the majority of these motifs may be false positive G4 sequences [21,22].

In this contribution, we present more elaborate computational models than regular expression to represent G4 motifs, employing hidden Markov models (HMMs). HMMs are so flexible in modeling linear dependence that they are widely used in bioinformatics including protein secondary structure prediction [23,24] and sequence motif search [25]. To model G4 motifs, we provide four HMM-based models from the viewpoint of the number of hidden states that describe G-runs and loops, and compare with each other in three computational experiments. The first preliminary experiment in predicting G-run regions in a set of 100 real G4 sequences in the literature [20] indicates that each HMM-based model can represent actual G-run regions well. The subsequent experiment in discriminating real and shuffled G4 sequences by using HMMs shows that the models considering detailed distributions of G-run and loop lengths can outperform the simple probabilistic extension of regular expression. In the third test with statistical analysis in discriminating highly likely G4 structures from putative G4 motifs in the human pre-mRNA sequences [26], the results show that the HMM-based model that can represent elaborate length distribution of G-run regions outperforms the other three models presented in this work. Moreover, the above model can be specialized to detect G4 sequences whose functional roles are expected to be involved in DNA transcription. Finally, this model in conjunction with pattern search is applied to G4 screening in the whole human genome, producing a considerably smaller number of G4 candidates with statistical significance than that of G4 sequences predicted by pattern matching alone.

Here we would like to emphasize the significance of our research findings as follows:

• As compared with the regular expression-based approach, our method can assess G4 motifs quantitatively by a probabilistic measure. Indeed, G4 motifs can be detected first by the "discrete" regular expression-based method and then may be scored to judge their thermodynamic stability using energy parameters for G4 structures. However, to the best of our knowledge, elaborate energy parameters for G4 structures have not been available so far. Under these circumstances, probabilistic models including HMMs are useful in not only evaluating predictions quantitatively but also training the model parameters from experimentally verified data.

• Our results show that HMM-based models are statistically reliable enough to detect a more specified motif among general G4 structures in genomic sequences, narrowing down potential G4 sequences predicted by the existing pattern matching method. This means that the combination of the regular expression-based approach and our probabilistic method will help reduce expensive and laborious wet-lab experiments more than the regular expression method alone will do to exhaustively analyze a given set of G4 motifs of interest. We believe that our research findings can boost understanding of functional roles of G4 structures in genomes, as well as helping to design therapeutic drugs that target specific G4 structures.

## Results and discussion

We develop four HMMs to see how well the models can represent real G4 sequences and can reduce false positive G4 sequences from putative ones. To put it simply, the HMMs developed have four sets of hidden states for G-runs linked by three sets of hidden states for loops (see Methods for details of HMMs). In addition, the parameters of HMMs were trained by experimentally verified data in the literature [20].

### Predicting G-run regions

Stegle *et al*. [20] provide a dataset of 260 G4 structures, which were experimentally verified with varying salt concentrations. Note that the corresponding sequences are of the form *G*^+^*N*^∗^*G*^+^*N*^∗^*G*^+^*N*^∗^*G*^+ ^in regular expression. In our test, we used 100 sequences out of 260 because the original dataset contains duplicate sequences with different salt concentrations.

We predicted G-run regions of each sequence in the dataset using the Viterbi algorithm for each HMM. Evaluation measures that we used are TP (true positive), the number of correctly predicted Gs in G-runs, FP (false positive), the number of incorrectly predicted Gs that are not in G-runs, and FN (false negative), the number of correct Gs in G-runs that are not predicted. In addition, the following measures were calculated:

$$\text{SEN}=\frac{\text{TP}}{\text{TP}+\text{FN}},\;\;\;\;\;\text{PPV}=\frac{\text{TP}}{\text{TP}+\text{FP}},\;\;\;\text{F}=\frac{2\times \text{SEN}\times \text{PPV}}{\text{SEN}+\text{PPV}},$$

where SEN, PPV and F denote sensitivity, positive predictive value and F-measure, respectively.

Results of predicting G-run regions are shown in Table 1 where 10-fold cross validation was carried out for training and testing. It should be noted that training was carried out using the Baum-Welch algorithm for each HMM. As the table shows, every model achieves high PPV, meaning that few FPs are found in the predictions. The HMM-based model 1 achieves perfect sensitivity, although it produces the most FPs. This tells us that the model 1 considers all consecutive G regions as G-runs. On the other hand, the models 3 and 4 yield few FPs, but the model 3 outputs the most FNs, degrading sensitivity. All things considered, the model 1 seems better to predict G-run regions.

**Table 1:** Results of predicting G-run regions in 100 real G4 sequences verified experimentally in [20].

HMM	TP	FP	FN	SEN	PPV	F	Time (s)
Model 1	**1196**	3	**0**	**1.000**	0.998	**0.999**	**0.008**
Model 2	1113	2	83	0.931	0.998	0.963	0.014
Model 3	886	**1**	310	0.741	**0.999**	0.851	0.017
Model 4	1080	**1**	116	0.903	**0.999**	0.949	0.025

In this test, we evaluated predictive performance of four HMM-based models using 10-fold cross validation. Time shows the total execution time to run the Viterbi algorithm on a machine with Intel Core i7-3820 3.60GHz CPU and 3.00GB RAM. The best performance is shown in boldface.

### Discriminating G4 sequences

We first investigate the discriminative performance of the four HMM-based models between real and shuffled G4 sequences. More specifically, we first randomly split the set of 100 real G4 sequences in Stegle et al.'s dataset [20] into two sets of 50 positive sequences, where one set is for training and the other is for validation. Next, a set of 50 negative sequences for validation was created by doing trinucleotide shuffling [27] of 50 positive sequences in the validation set. Note that use of trinucleotide shuffling comes from the observation that G4 structures often have at least three consecutive Gs as each G-run to make their structures stable. In total, we have 100 sequences in the validation set where 50 sequences are positive and the other 50 sequences are negative.

Here we elaborate on the evaluation of the full probability *P *(*x | θ*) of a sequence *x *given an HMM with a set *θ *of parameters, which can be calculated by the forward algorithm. Note that the parameters of each HMM were trained on the training set of 50 G4 real sequences in the training set. Since the full probability values for HMMs are usually small and the naïve log likelihood scores are strongly length dependent, we used the log-odds score *L*(*x*) relative to a random model *R *defined by

$$L(x)=\log\frac{P(x|\theta )}{P(x|R)}=\frac{P(x|\theta )}{\prod_{\beta \in \{A,C,G,T\}}q_{\beta }},$$

where *q_β _*is the frequency of a base *β *∈ {*A, C, G, T *} in all training sequences. Finally, we converted the log-odds score of the sequence *x *into the Z-score over all validation sequences calculated by

$$Z\left(x\right)=\frac{L\left(x\right)-\bar{L}}{s},$$

where $\bar{L}$ and *s *denote the average and the standard deviation, respectively, of all validation sequences.

**Figure 2:**
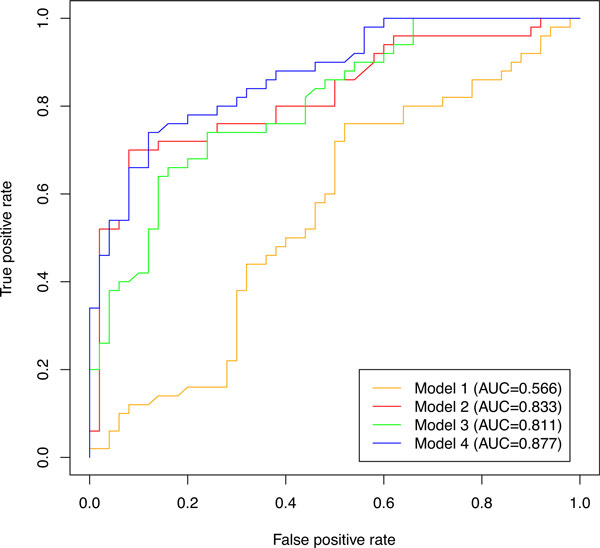
**Receiver operating characteristic (ROC) curves for HMM-based models**. This graph shows the discriminative power of each model between positive and negative G4 sequences created from Stegle et al.'s dataset [20]. Area under the ROC curve (AUC) is shown in the legend.

With the total number of 100 positive and negative sequences stated above, we computed Z-scores for log-odds scores of validation sequences in each HMM model, and drew receiver operating characteristic (ROC) curves and calculate area under the ROC curve (AUC) to judge the discriminative power. Figure 2 shows the ROC curves as well as AUC for each HMM-based model, indicating that the models 2, 3 and 4 perform good discrimination, whereas the model 1 yields the worst AUC. This means that the model 1 that can be considered as a simple probabilistic extension of regular expression is not enough to discern real G4 sequences from shuffled ones, and the models that considers detailed distributions of G-run and loop lengths can outperform the regular expression-based methods.

### Reducing potential G4 sequences in database

We next aimed to screen highly likely G4 structures out of putative G4 sequences predicted by pattern matching. To this end, we used G-Rich Sequence Database version 2.0 (GRSDB2) [26] that contains information on composition and distribution of putative G4 sequences mapped in the eukaryotic pre-mRNA sequences. We should notice that the sequence data stored in this database are computed by the regular expression-based approach named QGRS Mapper [17,18]. In this test, we retrieved 12,930 putative non-overlapping G4 sequences in 50 pre-mRNA genes with RefSeq status validated in the human genome (see Table 2). This time the parameters of each HMM were trained on the full dataset of 100 G4 sequences in Stegle et al.'s dataset.

**Table 2:** Human genes that include putative non-overlapping G4 sequences used in our experiments.

Gene symbol	# putative G4s	Length (nt)	Gene symbol	# putative G4s	Length (nt)
AHNAK	762	113317	MAWBP	204	50268
ARS2	105	13551	MGC3207	72	9751
BPGM	102	33014	MGC4707	815	227682
C14orf138	22	7942	NGFRAP1	19	1734
CCM2	373	76283	NT5C3	120	48668
CNOT4	309	148303	PHF14	336	195730
COMMD6	18	11871	PLEKHH1	295	56220
DIP2A	471	109711	PPP1R9A	742	386048
DKFZp761I2123	161	20951	RAB37	457	76205
DNAJA5	76	29372	RAP1B	130	49723
EGFR	695	188307	RGS6	2135	630822
ERCC1	118	14306	SEMA5B	786	119412
FLJ20097	232	126686	SF1	78	14164
FMO3	57	26924	SLC37A3	303	64760
FOXM1	86	19455	SP8	29	4605
FPRL1	41	9327	SUNC1	109	41972
FUS	82	11648	SYNJ1	256	99205
GMFB	24	14536	TFEC	145	95597
HTF9C	69	5371	TJP2	283	81032
IFRD1	171	53022	TRAF7	273	22332
IMPDH1	176	17976	UPP1	108	19976
ITM2C	135	14343	USP42	26	56635
KIAA2010	174	52859	ZAP70	222	26293
KRIT1	105	47132	ZCCHC11	312	129797
LOC285989	86	14703	ZNF32	25	5020

These are actually to be pre-mRNAs compiled in the Guanine Rich Sequence Database version 2.0 (GRSDB2) [26]. Note that # putative G4s indicates the number of G4 candidates stored in the database.

**Figure 3:**
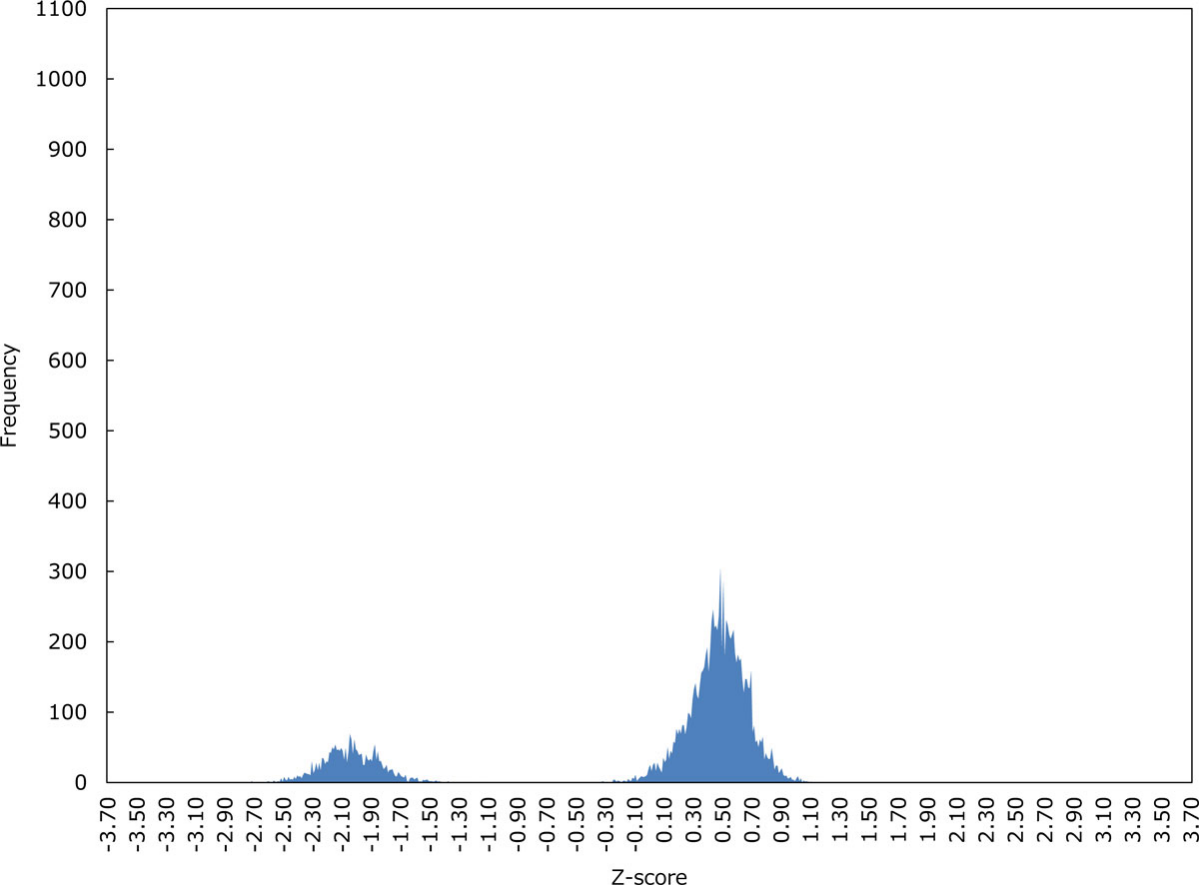
**The distribution of Z-scores in the HMM-based model 1**. Z-scores were calculated over 12,930 putative G4 sequences in 50 pre-mRNA genes stored in G-Rich Sequence Database version 2.0 (GRSDB2) [26].

**Figure 4:**
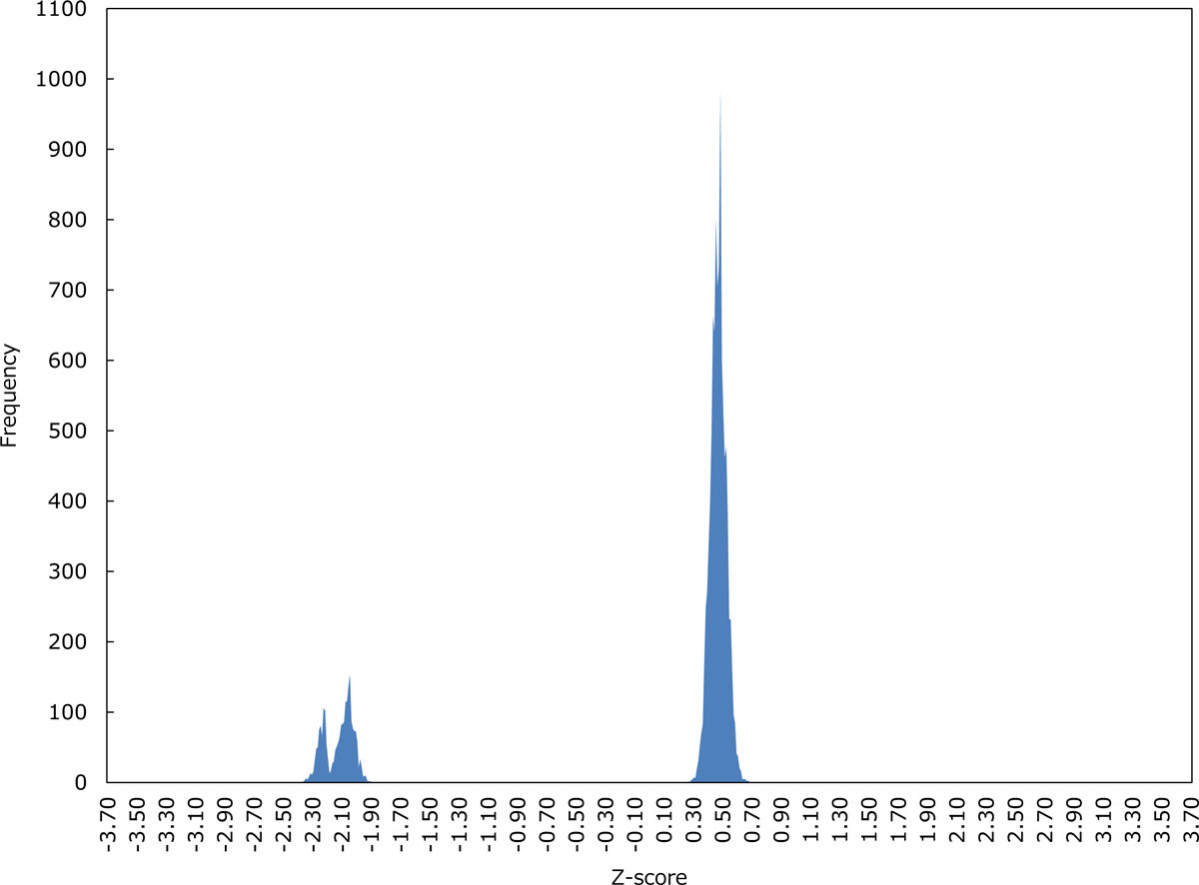
**The distribution of Z-scores in the HMM-based model 2**.

**Figure 5:**
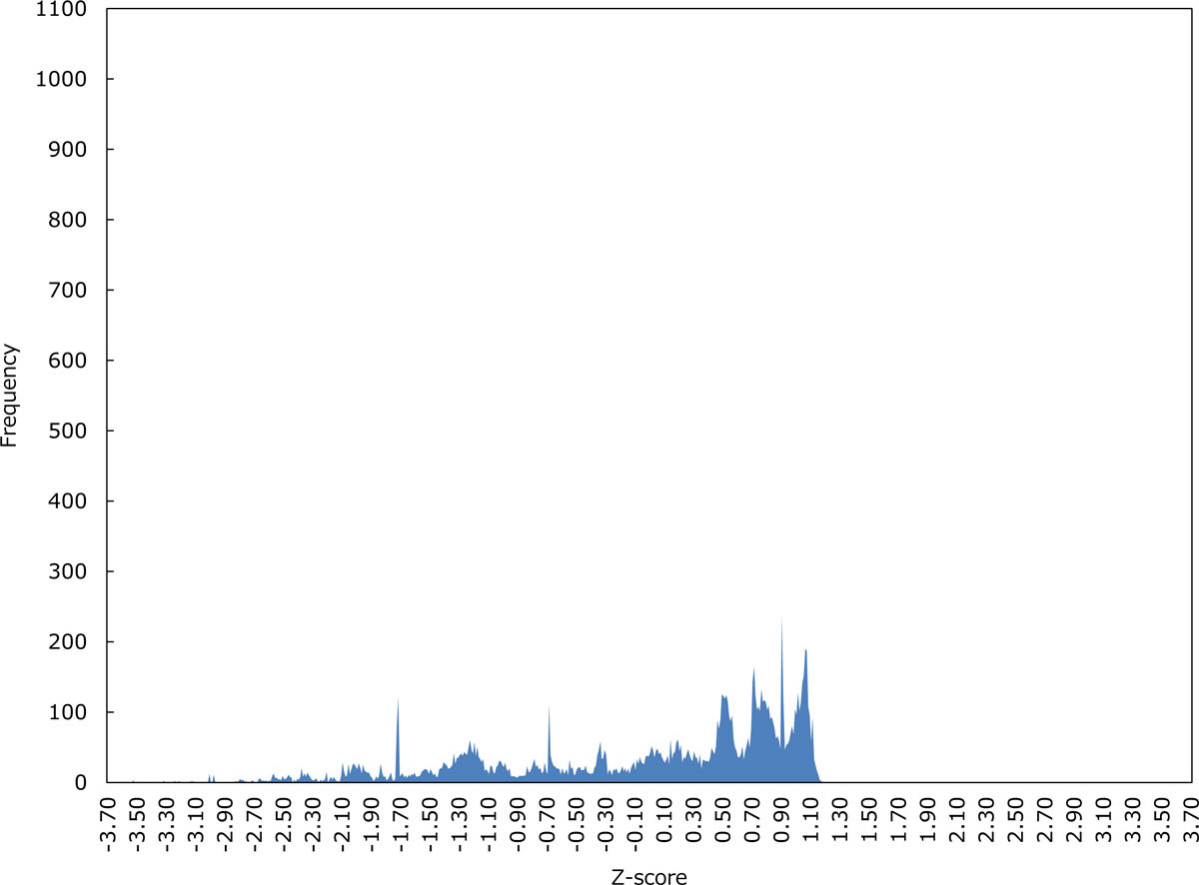
**The distribution of Z-scores in the HMM-based model 3**.

**Figure 6:**
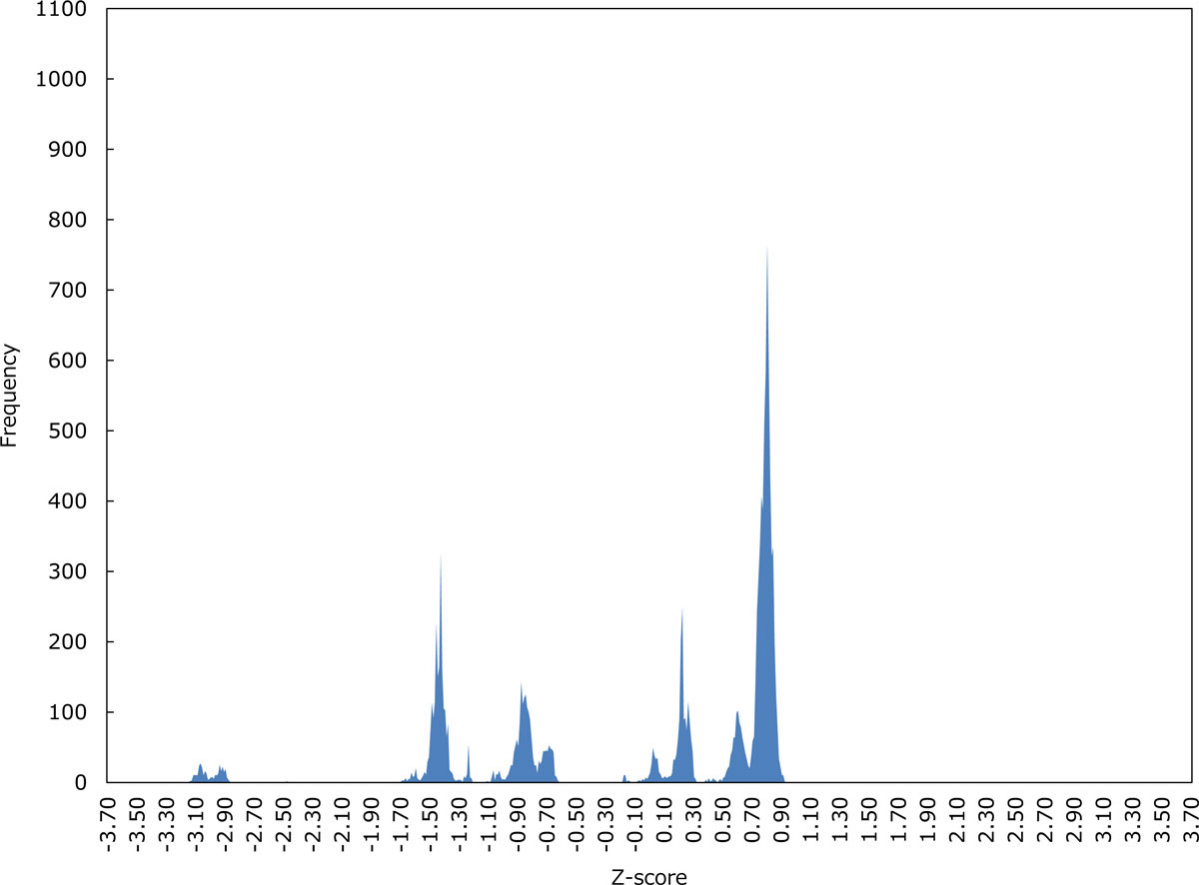
**The distribution of Z-scores in the HMM-based model 4**.

Figures 3-6 show the distributions of Z-scores of respective HMM-based models. We can see in Figures 3 and 4 that the distribution of Z-scores in the model 1 is very similar to that in the model 2. Looking more carefully into the shapes of these distributions, variance of each peak in the model 2 is smaller than that in the model 1. Moreover, the rightmost peak of higher Z-scores contains many putative G4 sequences in the model 2. In Figure 5, the frequency of Z-scores is roughly said to rise steadily from left to right and there seem to be a few peaks in the model 3. This means that unlike the models 1 and 2, we cannot separate the distribution clearly in the model 3. The distribution in the model 4 shown in Figure 6 has several peaks, indicating that we can separate the distribution into multiple groups.

**Figure 7:**
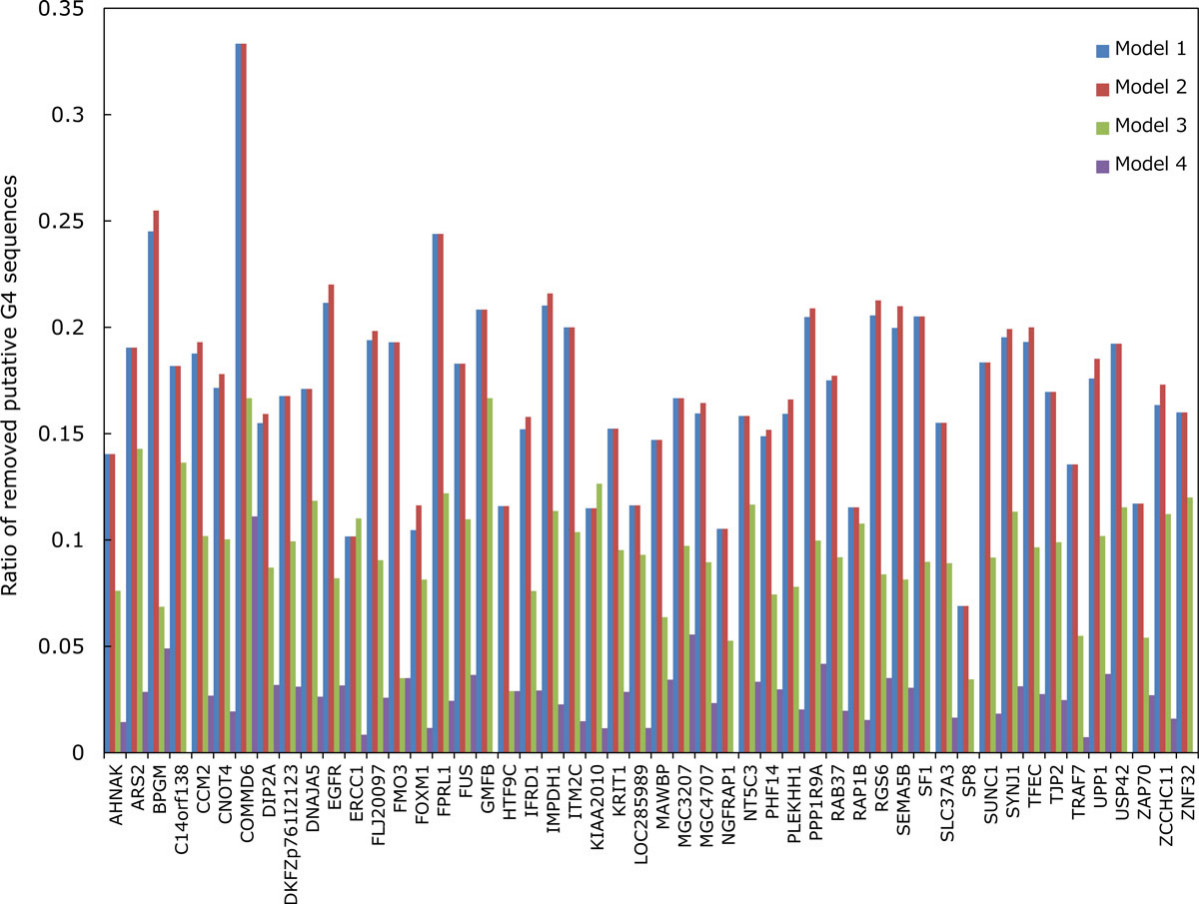
**The ratio of the number of putative G4 sequences in each gene reduced by the cutoff in each HMM-based model**. The cutoff is -1.645 of lower-tailed 5% point of the standard normal distribution. Blue, red, green and purple bars correspond to the models 1, 2, 3 and 4, respectively.

Let us now evaluate the Z-scores statistically. When we cut out Z-scores under -1.645 of lower-tailed 5% point of the standard normal distribution, the distribution in the model 2 can be perfectly separated into two groups as shown in Figure 4. In contrast, Figures 5 and 6 show that we cannot separate the distributions in the models 3 and 4 perfectly by the significant Z-score threshold. Note that in the model 1 the leftmost peak includes this cutoff and thus the distribution can only be partly separated (see Figure 3). To investigate the difference in the number of G4 candidates that can be reduced by the cutoff between four HMMs, we drew the graph that shows the ratio of putative G4 sequences reduced by the threshold in each gene (see Figure 7). As we can see, the model 2 reduces the most G4 sequences, whereas the model 4 leaves the most. Therefore, we will compare the model 2 with the model 4 below.

### Functional analysis of putative G4 sequences

Here we focus on the putative G4 sequences with significant Z-scores in the HMM-based models 2 and 4, computing the ratio of the number of G4 sequences in a gene *X *to the length of that gene, called G4 density for the gene, defined as

$$D\left(X\right)=\frac{G\left(X\right)}{\left|X\right|},$$

where *G*(*X*) is the number of G4 sequences in the gene *X *and *|X| *shows the length of *X*. For the original G4 candidates in the GRSDB2 database and their reduced G4 sequences computed by the HMM-based models 2 and 4 with the cutoff of lower-tailed 5% point of the standard normal distribution, we calculated G4 density of each gene and converted it into the Z-score in each case. It should be noted that the Z-scores were calculated over all genes in each case. We should also notice that the point here is to make clear which gene can be considered to have significantly many G4 sequences.

Tables 3 and 4 show the results of G4 density in the models 2 and 4, respectively. These results indicate that the model 2 can narrow down the number of genes by one in statistical interpretation as compared with the reference candidates and reductions by the model 4 with the significant Z-score threshold. Looking more carefully into the genes with significantly many G4 sequences computed by the model 2, they are likely to be involved in transcriptional regulation (see Table 5). Therefore, the HMM-based model 2 can be helpful in detecting G4 sequences whose functional roles are related to DNA transcription.

**Table 3:** G4 density of each gene computed from the results of the HMM-based model 2.

Gene symbol	*Z*(*D_pred_*)	*Z*(*D_ref _*)	Gene symbol	*Z*(*D_pred_*)	*Z*(*D_ref _*)
HTF9C	**2.914**	**2.753**	FPRL1	-0.275	-0.138
TRAF7	**2.601**	**2.540**	MGC4707	-0.407	-0.417
NGFRAP1	**2.298**	**2.107**	KIAA2010	-0.438	-0.515
IMPDH1	1.453	**1.708**	TJP2	-0.444	-0.447
ITM2C	1.395	1.578	EGFR	-0.452	-0.379
ZAP70	1.365	1.247	IFRD1	-0.517	-0.538
ERCC1	1.347	1.180	RGS6	-0.537	-0.484
DKFZp761I2123	0.944	0.987	RAP1B	-0.677	-0.747
ARS2	0.896	1.009	BPGM	-0.681	-0.585
MGC3207	0.848	0.884	C14orf138	-0.695	-0.694
SP8	0.733	0.513	DNAJA5	-0.743	-0.756
AHNAK	0.700	0.659	SUNC1	-0.753	-0.753
FUS	0.689	0.767	NT5C3	-0.771	-0.798
SEMA5B	0.470	0.610	SYNJ1	-0.775	-0.759
LOC285989	0.457	0.359	ZCCHC11	-0.806	-0.819
RAB37	0.364	0.410	KRIT1	-0.845	-0.879
UPP1	0.154	0.208	CNOT4	-0.915	-0.929
SF1	0.143	0.242	FMO3	-0.917	-0.917
PLEKHH1	0.142	0.154	PPP1R9A	-0.991	-0.984
ZNF32	0.066	0.062	FLJ20097	-1.012	-1.015
SLC37A3	-0.025	-0.041	PHF14	-1.017	-1.054
CCM2	-0.028	0.031	GMFB	-1.076	-1.077
FOXM1	-0.044	-0.129	TFEC	-1.113	-1.123
DIP2A	-0.162	-0.173	COMMD6	-1.194	-1.123
MAWBP	-0.221	-0.253	USP42	-1.448	-1.484

The column of *Z*(*D_pred_*) shows the Z-score for G4 density *D_pred _*of each gene that contains G4 sequences reduced by the cutoff of lower-tailed 5% point of the standard normal distribution. The column of *Z*(*D_ref _*) indicates the Z-score for G4 density *D_ref_* of each gene that contains original G4 candidates in the GRSDB2 database. A significantly large Z-score at the significance level 0.05 is shown in boldface.

**Table 4:** G4 density of each gene computed from the results of the HMM-based model 4.

Gene symbol	*Z*(*D_pred_*)	*Z*(*D_ref _*)	Gene symbol	*Z*(*D_pred_*)	*Z*(*D_ref _*)
HTF9C	**2.732**	**2.753**	MAWBP	-0.272	-0.253
TRAF7	**2.613**	**2.540**	EGFR	-0.393	-0.379
NGFRAP1	**2.199**	**2.107**	MGC4707	-0.420	-0.417
IMPDH1	**1.712**	**1.708**	TJP2	-0.452	-0.447
ITM2C	1.608	1.578	RGS6	-0.501	-0.484
ZAP70	1.237	1.247	KIAA2010	-0.505	-0.515
ERCC1	1.224	1.180	IFRD1	-0.548	-0.538
ARS2	0.995	1.009	BPGM	-0.616	-0.585
DKFZp761I2123	0.967	0.987	C14orf138	-0.675	-0.694
MGC3207	0.801	0.884	RAP1B	-0.744	-0.747
FUS	0.734	0.767	SUNC1	-0.752	-0.753
AHNAK	0.679	0.659	DNAJA5	-0.763	-0.756
SEMA5B	0.593	0.610	SYNJ1	-0.770	-0.759
SP8	0.563	0.513	NT5C3	-0.811	-0.798
RAB37	0.417	0.410	ZCCHC11	-0.817	-0.819
LOC285989	0.382	0.359	KRIT1	-0.888	-0.879
SF1	0.286	0.242	CNOT4	-0.930	-0.929
UPP1	0.180	0.208	FMO3	-0.930	-0.917
PLEKHH1	0.157	0.154	PPP1R9A	-1.001	-0.984
ZNF32	0.101	0.062	FLJ20097	-1.021	-1.015
CCM2	0.023	0.031	PHF14	-1.063	-1.054
SLC37A3	-0.032	-0.041	GMFB	-1.068	-1.077
FOXM1	-0.114	-0.129	TFEC	-1.130	-1.123
FPRL1	-0.142	-0.138	COMMD6	-1.174	-1.123
DIP2A	-0.188	-0.173	USP42	-1.486	-1.484

A significantly large Z-score of G4 density at the significance level 0.05 is shown in boldface.

**Table 5:** Information on genes that have significantly many putative G4 structures.

Gene symbol	Length (nt)	Gene ontology
HTF9C	5371	**Function: **RNA binding, methyltransferase activity, nucleotide binding. **Process: **metabolic process.
TRAF	22332	**Function: **ligase activity, metal ion binding, protein binding, ubiquitin-protein, ligase activity, zinc ion binding. **Process: **activation of MAPKKK activity, positive regulation of MAPKKK cascade, protein ubiquitination, regulation of apoptosis, regulation of transcription, DNA-dependent, transcription. **Component: **ubiquitin ligase complex.
NGFRAP1	1734	**Function: **metal ion binding, molecular function. **Process: **apoptosis, biological_process, multicellular organismal development. **Component: **cellular_component, nucleus.
IMPDH1	17976	**Function: **IMP dehydrogenase activity, catalytic activity, metal ion binding, oxydoreductase activity, potassium ion binding. **Process: **GMP biosynthetic process, GTP biosynthetic process, lymphocyte proliferation, metabolic process, purine nucleotide biosynthetic process, response to stimulus, visual perception.

Note that in the gene ontology column, the words "molecular_function," "biological_process" and "cellular_component" are used to show that details are unclear.

### Applying HMM to whole human genome

The third experiment stated above focuses only on pre-mRNA sequences in the human genome, leaving further potential G4 sequences over the whole genome. Thus, we demonstrate here how many potential G4 sequences the regular expression-based method can detect in the whole human genome and how many our method can reduce.

**Figure 8:**
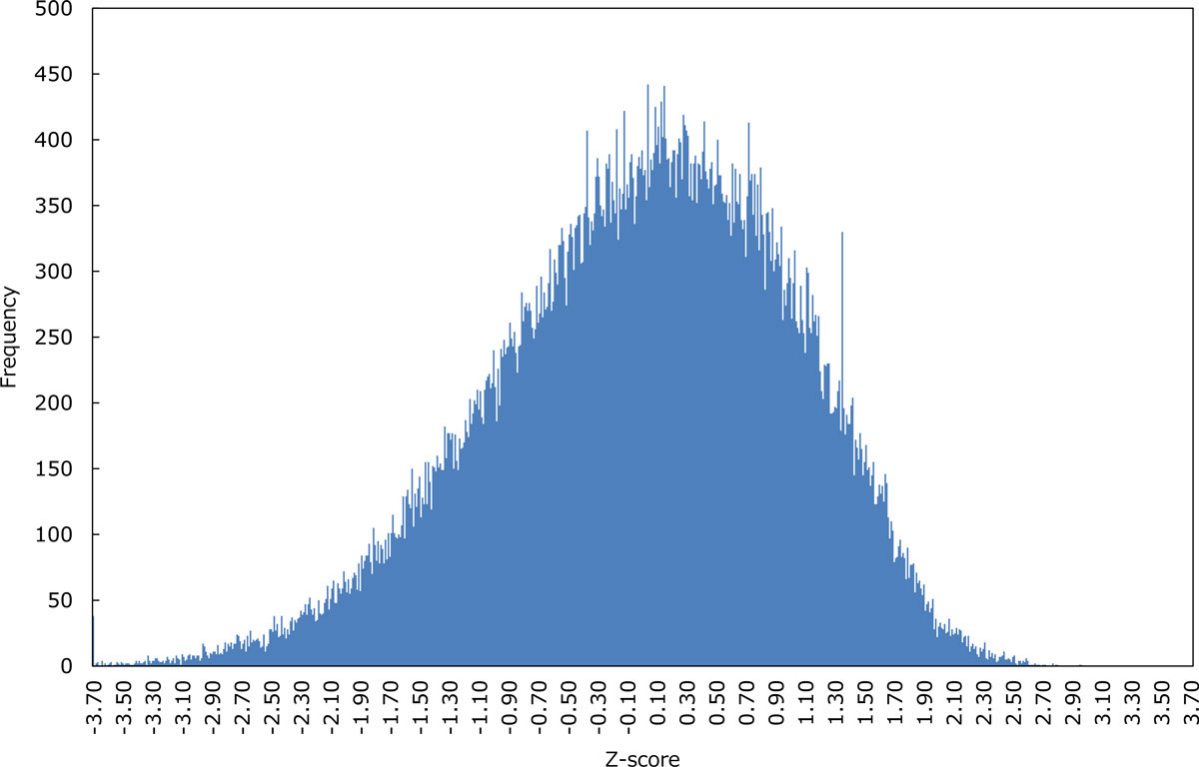
**The distribution of Z-scores in the HMM-based model 2 on the human genome stored in the UCSC Genome Browser database **[28]. Z-scores were calculated over 100,332 putative G4 sequences detected by the regular-expression based method.

The human genomic sequence named hg19 was retrieved from the UCSC Genome Browser database [28], where we used 22 regular chromosomes along with X, Y and M chromosomes. In the regular expression *G*^+^*N*^∗^*G*^+^*N*^∗^*G*^+^*N*^∗^*G*^+ ^that we used as prefilter, the length of a G-run is between three and five and that of a loop is between one and seven. This assumption comes from the majority of existing pattern matching-based methods [11]. After running regular expression-based search for non-overlapping G4 motifs on the set of genomic sequences, we attempted reducing the resulting putative G4 sequences by using the HMM-based model 2. More specifically, we calculated Z-scores over all putative G4 sequences as in the third experiment, and discriminated the G4 sequences with Z-scores by two cutoffs that indicate lower-tailed and upper-tailed 5% points of the standard normal distribution, respectively. Figure 8 shows the distribution of Z-scores in the model 2, whose shape is similar to that of the normal distribution. In Table 6, we can see that the run-time of regular expression-based search followed by HMM screening is short enough to handle genomic sequences of huge size. Comparing the ratios of reduced G4 sequences using the two cutoffs, discrimination by upper-tailed 5% point yields more drastic reductions than that by lower-tailed 5% point. Accordingly, the remaining G4 candidates with significantly high Z-scores reduced from those detected by pattern matching are expected to be highly likely to form G4 structures in vitro and in vivo.

**Table 6:** Comparison of the number of G-motifs in the human genome between use of regular expression (RE) alone and that of HMM (model 2) together with RE.

Model	# G4 motifs	% Reduction	Time
RE	100332	N/A	2m 5.296s
RE+HMM with cutoff 1	94272	6.040	2m 5.296s + 22.245s
RE+HMM with cutoff 2	3285	96.726	2m 5.296s + 22.245s

Note that RE+HMM represents the RE-based search followed by HMM screening. In addition, % Reduction shows how many G4 motifs the RE+HMM model can reduce from the results of RE only. We used two cutoffs to remove less significant potential G4s, where the cutoff 1 of -1.645 and the cutoff 2 of 1.645 mean lower-tailed and upper-tailed 5% points of the standard normal distribution, respectively. Run-time was calculated on a machine with Intel Core i7-3820 3.60GHz CPU and 3.00GB RAM.

## Discussion

From our experimental results, the following two points on the constitution of HMMs become clear:

• Increasing the hidden states for representing G-runs in an HMM can lead to small variance of the probability distribution over input sequences given the model.

• Increasing the hidden states for describing loops can make the HMM flexible.

The first point can be explained by Figures 3 and 4, while Figure 5 gives a good account of the second point.

Here we will look closely at G-runs and loops in G4 sequences. Recall that G-run is a region of consecutive Gs involved in G-quartets and loop is a single strand consisting of arbitrary bases that connect G-runs in front and behind. Since the HMM-based model 2 as well as the model 4 is specialized to represent consecutive Gs, each G in G-runs will be strictly discriminated in the model, affecting the sharpness of the probability distribution over the set of input sequences. On the other hand, the model 3 has more hidden states that can represent any base, and thus it can output an arbitrary sequence in a more flexible framework and show multi-modal probability distribution. Viewed in this light, we may say that the model 4 has the broader distribution of Z-scores due to increase in hidden states for representing loops, and several groups of peaks because of increase in hidden states for describing G-runs (see also Figure 6). Although the different peaks in score distributions may tell us which potential G4 sequence actually forms G4 structure in vitro and/or in vivo, experimental verification in wet-labs is still awaited.

## Conclusions

We presented the HMM-based modelings for G4 motifs in anticipation of reducing false positive G4 sequences in genomic DNAs detected by simple pattern matching with regular expression. The discrimination test with the HMMs was indicative of high discriminative power of elaborate models between positive and negative G4 sequences. Our computational experiments with statistical analysis on potential G4 sequences in human genomes make it clear that the HMM-based model that considers detailed distribution of G-run length can discriminate well between G4 sequences that match the model and those that do not. Moreover, another experimental results suggest that the above HMM-based model can be specialized to detect genes whose functional roles are expected to be involved in transcription, which include significantly many G4 sequences. Furthermore, this model in conjunction with use of regular expression can detect a considerably smaller number of G4 candidates in the whole human genome with statistical significance. Therefore, we may reasonably conclude that the HMM-based approach together with the conventional pattern matching method can contribute to reducing costly and laborious wet-lab experiments to exhaustively analyze a given set of G4 motifs of interest.

In this work, we proposed the HMM-based models where each G-run has variable length. In contrast, applying HMMs that deal only with a specific fixed length of G-runs to genomic sequences may yield more accurate discrimination of G4 sequences. In addition, change of the training sequences that should be verified experimentally may have a certain effect on prediction results. In this sense, collaboration between in silico, in vitro and in vivo experiments will be even more important to advance functional analysis of G4 structures in genomes of various organisms.

## Methods

A G4 sequence comprises alternate G-runs and loops, which can be described as *G*^+^*N*^∗^*G*^+^*N*^∗^*G*^+^*N*^∗^*G*^+ ^in regular expression. In particular, the majority of existing pattern matching-based methods assume that the length of a G-run is between three and five and that of a loop is between one and seven [11]. To model the G4 motif by HMMs, we focus on which state of G-run and loop each base in a given sequence is decoded into. Advantages of use of HMMs can be summarized as follows:

• The most likely state path that corresponds to structural elements in a sequence can be predicted by the Viterbi algorithm.

• The probability of a sequence given the parameterized model can be calculated by the forward algorithm.

• Optimal probability parameters of the model can be estimated on a set of example sequences by the Baum-Welch algorithm.

**Figure 9:**
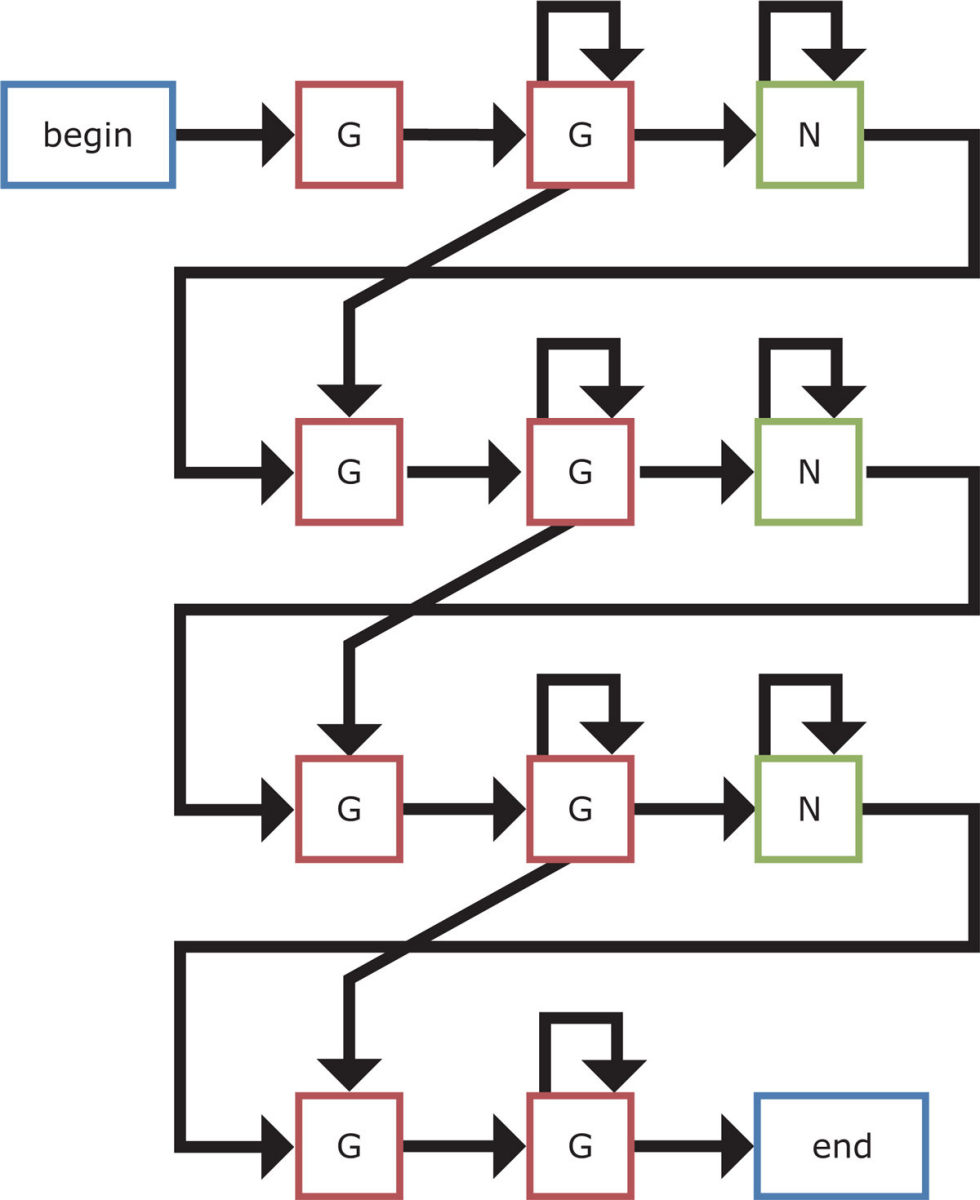
**The HMM-based model 1**. In the figure, a letter surrounded on four sides indicates a hidden state for emitting that letter. Specifically, "G" is the hidden state for G-runs; "N" is the hidden state for the interconnecting loops; "begin" state shows the start of the motif; and "end" state denotes the end of the motif. Note that the "begin" and "end" states actually generate no symbols. An arrow between two hidden states shows state transition. Note that a probability parameter is given to each transition. This model is designed as simple as possible, where at least two Gs are emitted from two states for each G-run and zero or more bases are allowed to emit from each loop state.

**Figure 10:**
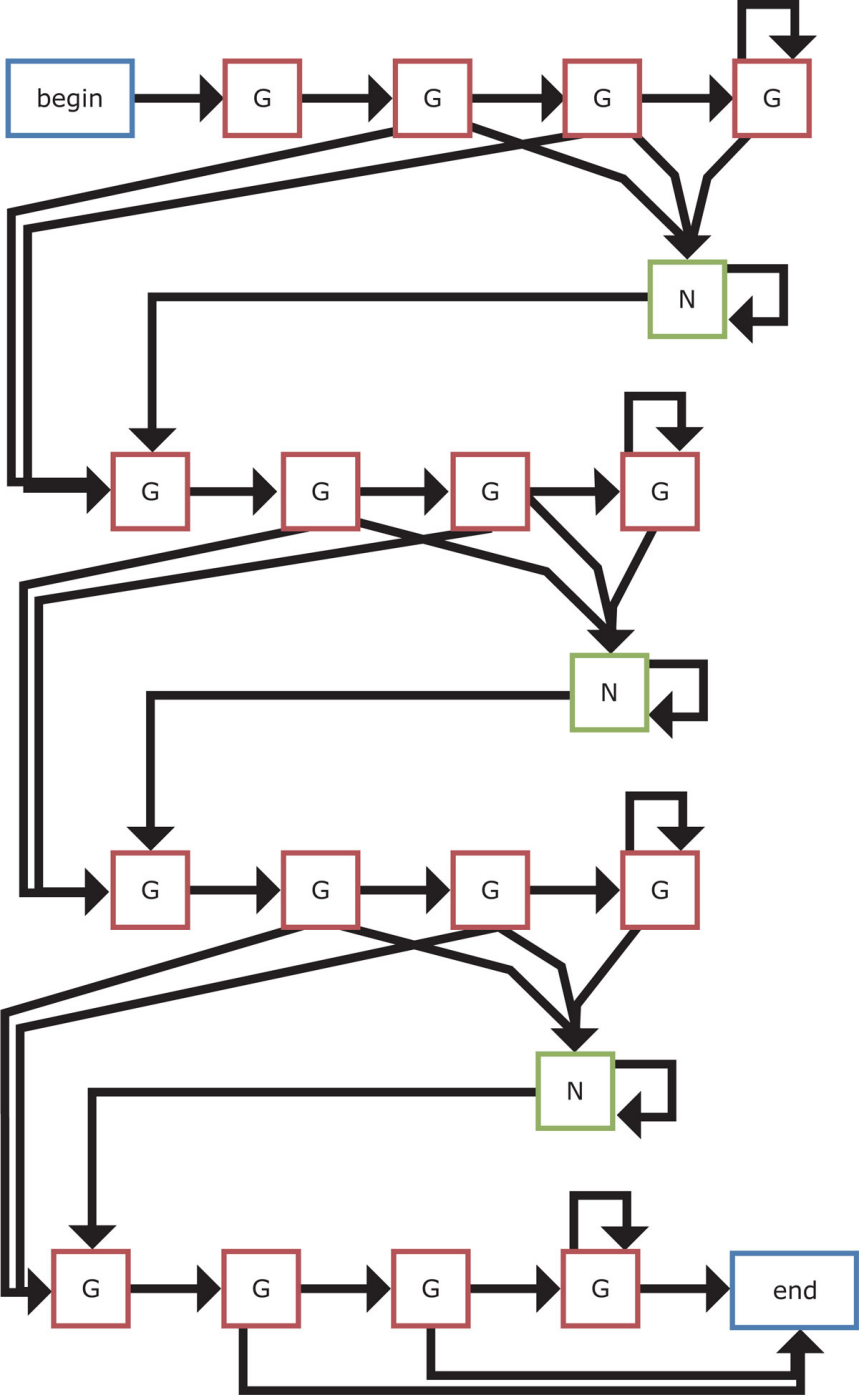
**The HMM-based model 2**. This model is an extension of the model 1 where hidden states for emitting G that correspond to each G-run increase to four.

**Figure 11:**
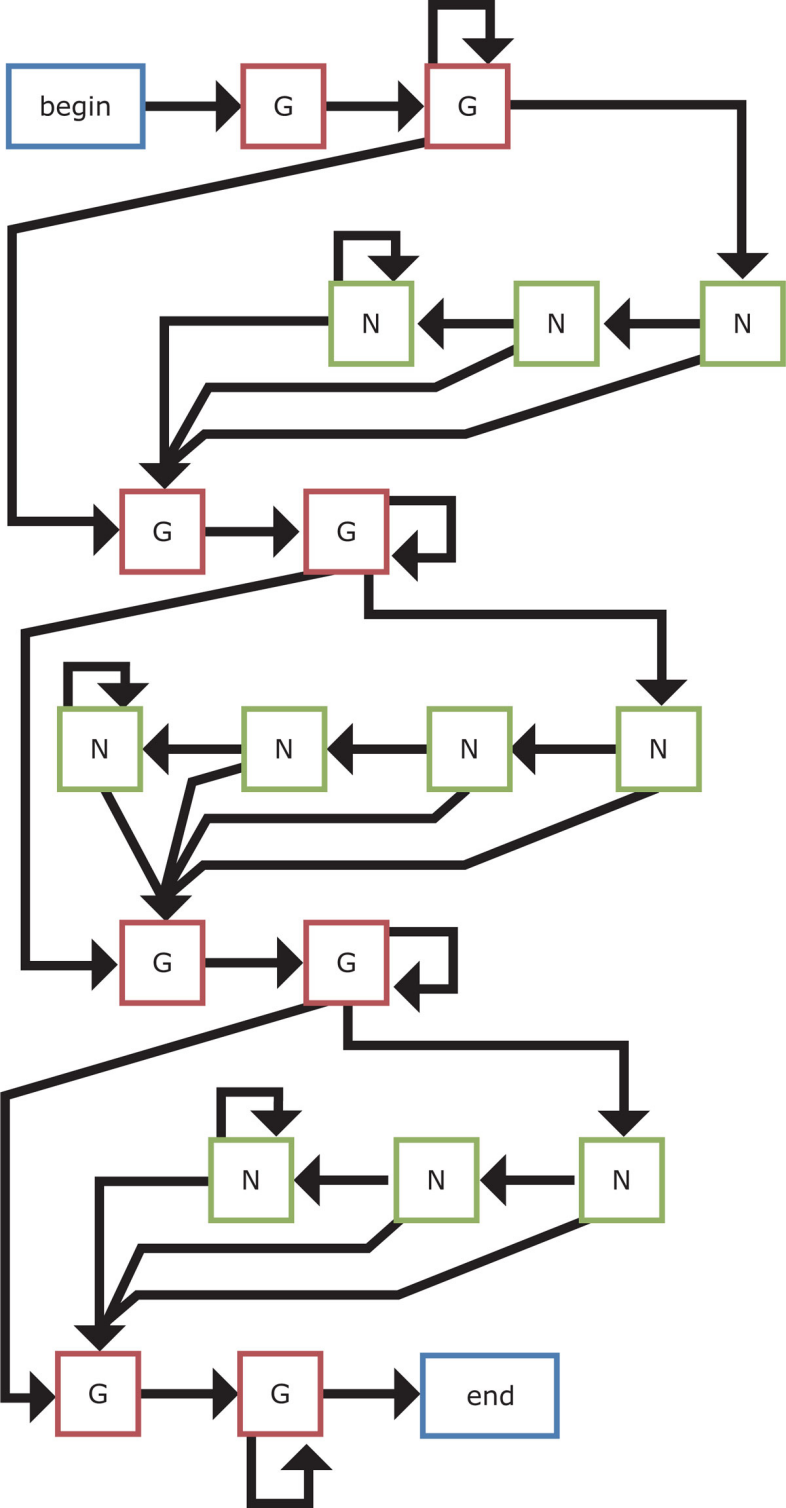
**The HMM-based model 3**. This model is another extension of the model 1 where hidden states for emitting any bases that correspond to each loop increase to three or four.

**Figure 12:**
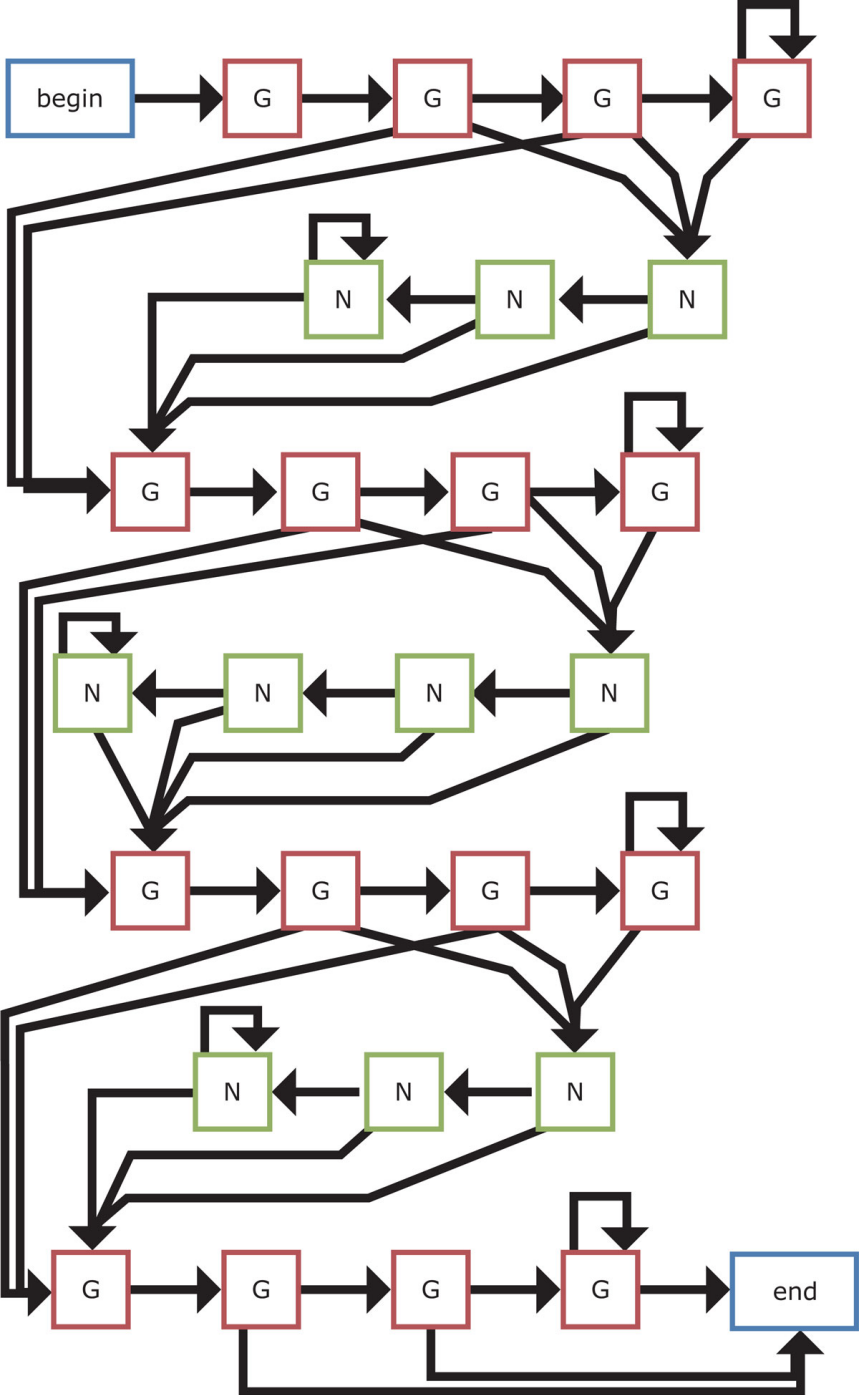
**The HMM-based model 4**. This model is the hybrid model of the models 2 and 3.

The four HMM-based models that we present in this work are shown in Figures 9-12. We first design the model 1 as simple as possible, where at least two Gs are emitted from two hidden states for each G-run and zero or more bases are allowed to emit from each hidden loop state. Then, an extension of the model 1 is considered, where hidden states for emitting G that correspond to each G-run increase to four. Another extension of the model 1 is also developed by increasing hidden states for emitting any bases that correspond to each loop to either three or four in turn. Note that this alternative number for increase in hidden states for loops is based on the training dataset [20]. Finally, we consider the hybrid model of the models 2 and 3 to be the model 4.

The Viterbi algorithm can compute the most probable state path of an HMM for a given sequence in *O*(*m*^2^*n*) time based on dynamic programming, where *m *is the number of hidden states in the HMM and *n *is the sequence length. The forward algorithm and the backward algorithm, which is analogous to the forward algorithm but differs in that a backward recursion starts at the end of a sequence, can compute the probability of a sequence given an HMM by dynamic programming with the same running time of the Viterbi algorithm. Finally, the Baum-Welch algorithm can calculate optimal parameters of an HMM given a set of training sequences, where the forward and backward algorithms are repeatedly used until the change in log likelihood of the sequences is less than some threshold. Details of the algorithms can be found in [25].

## Competing interests

The authors declare that they have no competing interests.

## Authors' contributions

MY designed and implemented the HMMs, and carried out all computational experiments. YK conceived the basic part of the work and wrote the manuscript. All authors read and approved the final manuscript.

## Declarations

Publication charges were supported by JSPS KAKENHI [#24700296 to YK]
